# Heritability of Boldness and Hypoxia Avoidance in European Seabass, *Dicentrarchus labrax*

**DOI:** 10.1371/journal.pone.0168506

**Published:** 2016-12-19

**Authors:** Sébastien Ferrari, Khaled Horri, François Allal, Alain Vergnet, David Benhaim, Marc Vandeputte, Béatrice Chatain, Marie-Laure Bégout

**Affiliations:** 1 Ifremer, Fisheries laboratory, Place Gaby Coll, L’Houmeau, France; 2 Ifremer, MARBEC UMR9190, Chemin de Maguelone, Palavas-les-Flots, France; 3 Laboratoire universitaire des sciences appliquées de Cherbourg, Normandie Univ, UNICAEN, LUSAC, Cherbourg, France; 4 Conservatoire National des Arts et Métiers. Intechmer, Cherbourg, France; 5 GABI, INRA, AgroParisTech, Université Paris-Saclay Jouy-en-Josas, France; 6 Ifremer, L3AS, Chemin de Maguelone, Palavas-les-Flots, France; Xiamen University, CHINA

## Abstract

To understand the genetic basis of coping style in European seabass, fish from a full factorial mating (10 females x 50 males) were reared in common garden and individually tagged. Individuals coping style was characterized through behavior tests at four different ages, categorizing fish into proactive or reactive: a hypoxia avoidance test (at 255 days post hatching, dph) and 3 risk-taking tests (at 276, 286 and 304 dph). We observed significant heritability of the coping style, higher for the average of risk-taking scores (h^2^ = 0.45 ± 0.14) than for the hypoxia avoidance test (h^2^ = 0.19 ± 0.10). The genetic correlations between the three risk-taking scores were very high (r_A_ = 0.96–0.99) showing that although their repeatability was moderately high (r_P_ = 0.64–0.72), successive risk-taking tests evaluated the same genetic variation. A mild genetic correlation between the results of the hypoxia avoidance test and the average of risk-taking scores (0.45 ± 0.27) suggested that hypoxia avoidance and risk-taking tests do not address exactly the same behavioral and physiological responses. Genetic correlations between weight and risk taking traits showed negative values whatever the test used in our population *i*.*e*. reactive individual weights were larger. The results of this quantitative genetic analysis suggest a potential for the development of selection programs based on coping styles that could increase seabass welfare without altering growth performances. Overall, it also contributes to a better understanding of the origin and the significance of individual behavioral differences.

## Introduction

Recent years have seen a growth of interest towards the causes and consequences of consistent differences in individual behavior over time or contexts constituting so-called “coping styles” or “personality” [1–4]. It has been clearly identified that, within species, individuals may react differently to the same situation. This inter-individual variability is generated by a collection of correlated physiological and behavioral responses, resulting in only a limited number of behavioral phenotypes. Coping style covers numerous traits such as boldness and shyness, avoidance of novelty, exploration, activity, aggressiveness and sociability [5]. Various behavioral models reflecting coping strategies exist for mammals, birds and teleosts such as cichlids, salmonids, sticklebacks and a large number of tropical fish (reviewed in [6] and [7]). This inter-individual variability in behavioral and physiological responses is conserved between taxa, suggesting it is genetically determined. A better understanding of this inter-individual variation is essential to improve our knowledge of the adaptive value of behavior [1, 8].

Individuals from a fish population can be clustered into two main categories, based on their predisposition to take risks: bold and shy [9]. Usually, boldness is associated with a proactive strategy whereas shyness is associated with a reactive strategy. Proactive individuals tend to engage in active avoidance or cope with stressful stimuli [10, 11] through a “fight or flight” response. Their behaviors differ from that of reactive individuals: 1) they are more aggressive/dominant [3, 12], 2) they show greater motivation to feed after transfer to a novel environment [6], 3) they rapidly approach new objects [3], 4) they take more risks (i.e. they are bolder) and are more likely to explore when exposed to novelty [13–16] and 5) they tend to develop behavioral routines [17, 18]. Physiologically, a proactive strategy is associated with a lower hypothalamus-pituitary-inter-renal (HPI) activity [6, 19–21] and a higher sympathetic reactivity [6] than the reactive strategy.

Usually, behavioral studies aiming at characterizing coping style are carried out on small samples, using time consuming individual tests. These tests do not make the characterization of a large number of individuals possible, while this is a prerequisite for quantitative genetics studies [22]. However, recent work developed tests characterizing fish coping style in small to medium size groups: hypoxia avoidance test (N = 24 in [3, 16, 23]) and risk taking test (N = 24 in [3], N = 30 in [16], N = 60 in [24], N = 500 in [25]). The first test, hypoxia avoidance test, was based on previous studies showing that fish with divergent stress coping styles react differently to hypoxic conditions [26, 27]. This suggests that measuring how fish escape hypoxic conditions can qualify individuals within a population with respect to stress coping style, taking activity, boldness and hypoxia tolerance into account [3, 7, 16, 23]. The second one, risk taking test, has been successfully used to characterize fish boldness and activity in numerous experiments [3, 16, 24–26, 28].

Genetic variability of behavioral variation in teleosts has been studied only by a few authors [29–31]. To go further in the understanding of the origin and the significance of individual behavioral differences, it is important to improve our knowledge on the genetic basis of coping style and of its interactions with environmental conditions. Heritability, the proportion of phenotypic variance that can be attributed to additive genetic variance is estimated by measuring the similarity between relatives [32, 33]. Genetics of fish personality traits and coping style is still in its infancy, but some studies have shown *Quantitative Trait Loci* (QTL, in zebrafish, *Danio rerio* [34]) or genetic variance or heritability of boldness [30, 35]. Heritability of stress response, particularly post-stress plasmatic cortisol has been mainly studied in salmonids [36–42] and recently in European seabass, *Dicentrarchus labrax* [43, 44]. Divergent strains on post-stress plasmatic cortisol level have been developed in rainbow trout [41, 42]. This divergent selection led to strains also divergent for numerous traits of interest related to behavior, physiology and neurophysiology, making rainbow trout a model species for behavioral selection in teleosts [6, 20, 45–50]. It has also been shown that farmed trout populations selected for growth display proactive behavior [51]. All these studies suggest a potential for selection of individuals based on their coping styles. Understanding the genetic basis of these traits is key to understand the evolution of populations in changing environmental contexts [52]. Especially, quantitative genetics approaches could be of great value, as genetic variance is the major factor underlying the capacity of a trait to evolve in a population [32]. Moreover, evaluating different component traits under a quantitative genetics framework may help understand which part of the observed covariation between traits is of genetic or environmental origin [53]. As it is acknowledged that coping style might be composed by multi-dimensional independent components [54] efficient methods to assess the quantitative genetic components of coping styles are needed.

European seabass is a major species for Mediterranean aquaculture, but also a model species for genetic studies in marine fish. Genetic variability of growth performance and sex ratio in this species has been well established [55–61], but no study exists concerning the genetic variability of behavioral traits linked to coping style, and the genetic correlations between coping style and production traits such as growth.

In this study, we coupled genetic analyses of growth with that of boldness and hypoxia avoidance, using microsatellites to perform parental assignment and identify families, a widely applied method in fish, which enables rearing numerous families in common garden and thus controlling environmental effects, which then cannot confound family effects [61]. The aims of this study were therefore 1) to estimate the heritability of coping style traits (herein boldness and hypoxia avoidance which encompasses the individual measure of both risk taking and hypoxia sensitivity) and 2) to estimate the genetic and phenotypic correlations between growth and these traits.

## Material and Methods

### Ethical approval

All procedures performed in studies involving animals were in accordance with the ethical standards of the institution and followed the European Directive 2010/63 UE; this study was conducted under the official national license of M.L. Bégout (17–010). All fish handling procedures (except just before behavioral tests) were conducted under anesthesia to minimize animal stress and suffering. Fish behavior was monitored daily, and humane endpoints (euthanasia by an excess of anesthetic dose) were planned in case fish would exhibit abnormal behavior (isolation from the group with slow and erratic swimming during two consecutive days). The sample size was determined according to simulations performed for heritability estimation protocols, where protocols with 50 sires and 30 offspring per sire were devised appropriate to estimate both genetic parameters for binary traits such as hypoxia avoidance [62] and genetic parameters for continuous traits [63]. The expected mortality rate, unavoidable in experiments with that many fish and lasting 218 days, was between 2 and 3%, and this is why 1536 individuals were tagged to obtain the expected sample size of 1500. However, the total observed mortality (6.9%) was in excess of this expectation, due to a short term disease outbreak (*Tenacibaculum maritimum*) just after tagging at 179 dph which was promptly cured by our veterinarian.

### Broodstock selection, mating and rearing conditions

Fish were produced according to a full factorial mating design from first generation domesticated West Mediterranean broodstock, combining 10 dams and 50 sires by *in vitro* fertilization, giving a theoretical number of 500 full-sib families. The number of parents and offspring sample size was optimized in order to have reliable heritability estimates, based on simulations performed earlier for binary traits [62] and continuous traits [22, 63]. Mating was carried out using the procedures described in [64]. After fertilization, eggs were pooled and reared in common garden following seabass rearing standards [65]. A random sample of offspring (13.2 ± 4.3 g mean body weight; N = 1536) were individually tagged at 179 days post hatching (dph) using PIT tags for longitudinal follow up (growth and behavioral analyses). At the time of tagging, they were fin-clipped (0.5 cm^2^ caudal fin sample) using scissors for DNA extraction, microsatellite genotyping and parental assignment. Tagged fish were stocked in a single 5 m^3^ tank within a recirculating bio-filtered system, with water at 21.9 ± 1.7°C, salinity of 37.9 and under a photophase of 12 hours. Fish were fed *ad libitum* by self-feeder using a standard aquaculture diet (Neo-grower, Le Gouessant, France) all along the experiment duration.

### Growth performances

The variable chosen to evaluate biological performances over time was the body weight (BW) and growth of all fish was followed from 179 dph to 397dph.

Biometric measurements were performed under anesthesia (Benzocaine, 200 ppm, after tranquilization in the rearing tank with 70 ppm Benzocaine) at 179, 200, 228, 256, 305, 325 and 397 dph. Two days before each biometric measurement, fish were fasted. At each biometric measurement, they were individually identified using a PIT tag reader and weighed to the nearest 0.1 g. At 397 dph, all fish were sacrificed by anesthetic overdose using 400 ppm of Benzocaine, weighed and dissected for identification of sex by visual inspection of the gonads following Menu et al., [66].

### Parental assignment

DNA extraction and the genotyping of twelve microsatellite markers were performed by LABOGENA-DNA, the French laboratory for livestock genotyping (ISO 17025 accredited, Jouy-en-Josas, France) for every sire, dam and offspring. Using these multilocus genotypes, fish parentage assignment was performed with VITASSIGN [67] following authors recommendations, with two allelic mismatches tolerated.

### Characterization of coping styles

#### Hypoxia avoidance test

**Fig 1:**
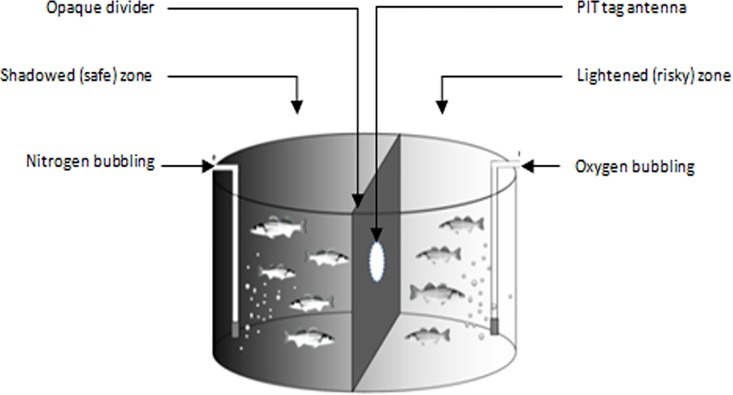
Scheme of the experimental set up used for the hypoxia avoidance test. Fish were gathered in one shadowed side of a circular tank (2.25 m in diameter, 5 m^3^ in volume) divided into two equal chambers by an opaque divider equipped with a PIT tag antenna surrounding a circular opening (12 cm in diameter). This enabled the monitoring of fish individual movement in a group situation without any disturbance. After 30 min of acclimation, nitrogen was bubbled in the shadowed chamber (called hypoxic chamber in the text) to reduce oxygen level and fish were allowed to freely move to the lit up chamber (called normoxic chamber) with normoxic conditions.

The hypoxia avoidance test was carried out at 255 dph on 1442 individuals held in a circular tank (5 m^3^, h: 144 cm, diameter: 210 cm, water height: 140 cm, Fig 1), which was strictly identical in size to the rearing tank. This tank was divided in two equal zones by an opaque divider with a circular opening (Ø 12cm) placed at 96 cm from the bottom, and equipped with a PIT tag antenna connected to a computer for data acquisition (Dorset, The Netherlands, adapted from Laursen et al., [23]). Each resulting chamber was considered to be a separate environment, individually equipped with an oxygen supply. All fish were placed in one chamber which was shadowed and subsequently considered as “safe” for the fish. The opening was closed for 30 minutes before the start of the test to allow fish acclimation. The test started with the switch from air and oxygen supply to nitrogen bubbling in the dark chamber containing the fish, progressively inducing hypoxic conditions during the experiment and further referred to as the "hypoxic” chamber. The second chamber of the tank, was maintained in normoxic conditions by oxygen supply (104.1% of saturation), and this hereafter called “normoxic” chamber was lit up to represent a “risky” area. Oxygen was monitored continuously using 3 oxymeters (Odeon®), two of them placed in the hypoxic chamber (one at the bottom and the other one under the water surface), and the third one placed in the middle of the water column of the normoxic chamber. The variables of interest were: individual time lapse to emerge from the hypoxia chamber to the normoxic chamber (emerg_hypo, in min); the individual fish escape order; the oxygen level in the hypoxic area at the first passage of each fish from the hypoxic to the normoxic chamber (O2mean, in percentage of saturation, calculated as the mean oxygen concentration between the two oxymeters); and the number of passages from the hypoxic to the normoxic chamber (NBhypo). The hypoxia test was stopped after two hours, when 10.3% oxygen saturation was reached in the hypoxic chamber (water temperature 21.9°C, salinity 37.9). A binary variable was also computed defining hypoxia-tolerant (HT) fish (Hypo_status = 0, when NBhypo = 0), also considered as reactive fish, and hypoxia avoider (HA) fish (Hypo_status = 1, when NBhypo ≥ 1), also considered as proactive fish (23).

#### Risk taking test

**Fig 2:**
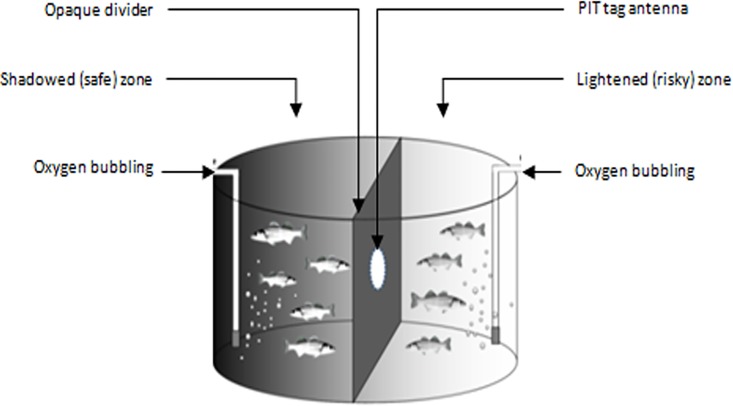
Scheme of the experimental set up used for the risk taking test sessions. Fish were gathered in one shadowed side (called safe chamber in the text) of a circular tank (2.25 m in diameter, 5 m^3^ in volume) divided into two equal chambers by an opaque divider equipped with a PIT tag antenna surrounding a circular opening (12 cm in diameter). This enabled the monitoring of fish individual movement in a group situation without any disturbance. After 30 min of acclimation, fish were allowed to freely move in the lit up chamber (called risky chamber in the text) with normoxic conditions during the next 24 hours with the usual photoperiod used during rearing.

Risk-taking behavior was assessed using the same experimental tank as for the hypoxia avoidance test, but maintaining optimal oxygen conditions in both chambers (Fig 2). Fish were transferred from their rearing tank into the shadowed chamber (hereafter called ‘safe chamber’ as opposed to the ‘risky chamber’). The opening was blocked for 30 minutes before the start of the test to allow fish acclimation. Individual fish passages from the safe chamber to the risky chamber through the opaque divider were monitored during 24h, with the usual photoperiod used during rearing (12D/12N) maintained in the risky chamber. This test was carried out three times (hereafter called sessions) at 276, 286 and 304 dph (RT1, RT2 and RT3 respectively) on the 1430 remaining individuals in order to assess consistency of the behavioral responses observed. The same procedure was used and environmental conditions were kept constant for all three sessions. The variables of interest were: the individual time-lapse to the first passage into the risky chamber (emerg_RT1, emerg_RT2,emerg_RT3,in min) used to assess risk taking level; the total number of passages through the opaque divider for each individual (NBRT1, NBRT2, NBRT3) used as a proxy of individual activity level; and the binary variables RT1_status, RT2_status and RT3_status defining risk takers (RT_status = 1, when NBRT ≥ 1) also referred as proactive, and risk avoiders (RT_status = 0 when NBRT = 0) further considered as reactive (23). Finally, new consolidated variables were calculated as means of each variable (NBRTmean, RT_status_mean) over the three sessions in order to assess the genetic variability of these variables.

#### Statistical analyses

Of the 1442 fish tested for Hypoxia Avoidance and consecutively the 1430 fish tested for Risk Taking, only 1243 could be unambiguously assigned to their parents, and among those 1155 were correctly sexed and were used for the statistical analyses. BW data were further analyzed as ln(BW) and back-transformed to original scale following [61].

Multi-trait sire models were applied to all traits, including BW at final biometric measurement, emerg_hypo, O2mean, NBhypo, Hypo_status, emerg_RT1, emerg_RT2, and emerg_RT3, NBRT1, NBRT2, NBRT3, RT1_status, RT2_status and RT3_status, to estimate (co) variance components:

$$Y_{ijk}=\mu +{Sex}_{i}+{sire}_{j}+\varepsilon_{ijk}$$

Where Y_ijk_ is the performance of the k^th^ individual, μ is the population mean, Sex_i_ is the fixed effect of sex (i = 1 for males, 2 for females), sire_j_ is the random additive genetic effect of sire j (j = 1 to 50) and ε_ijk_ is the random residual.

For all traits, the heritability, phenotypic correlations (r_P_) and genetic correlations (r_A_) amongst traits were estimated from the variance components using ASReml version 4.1 [68]. As the sire variance component accounts for ¼ of the additive genetic variance, heritability was estimated as follows:

$$h_{t}^{2}={4\sigma }_{ts}^{2}/\sigma_{tp}^{2}$$

Where h_t_^2^ is the heritability for the t^th^ trait, σ_ts_^2^ is the sire variance component for the t^th^ trait and σ_tp_^2^ the total phenotypic variance for the same trait [69].

For discrete binary traits, a generalized linear mixed effects model with binomial error structure and probit link function was used to estimate the sire variance component. In this case, the heritability on the underlying (probit) scale was estimated as follow:

$$h_{t}^{2}=4\sigma_{ts}^{2}/(\sigma_{ts}^{2}+\sigma_{\varepsilon }^{2})$$

where $\sigma_{\varepsilon }^{2}$ the residual variance on the underlying scale corresponding to the variance of the probit link which has a value of 1 [70].

For each trait, the significance of the random (sire) and fixed (sex) effects at a 5% alpha risk threshold was tested by likelihood ratio test (LRT) between nested models respecting marginality of the effects that are supposed to follow a *χ*^2^ distribution under the null hypothesis (type II tests; [71]). Diagnostics based on residuals were performed to assess the adequacy of the model and compliance to the underlying assumptions.

Finally, factorial ANOVAs were used to compare mean body weight of fish according to their sex and coping style. Chi-square tests were used to compare proportions of fish characterized as proactive or reactive in each test as well as sex ratio.

## Results

The dataset underlying our findings is available in the institutional public data repository (SEANOE: http://doi.org/10.17882/47080).

### Parentage assignment

DNA quality was unexpectedly poor in many individuals, and only 1267 individuals out of the 1442 fish tested for hypoxia tolerance showed DNA amplification, with only 1144 having a complete genotype for all 12 markers (90.2%). Parentage assignment revealed 329 full sib families out of the 500 possible ones, with 1 to 28 fish per full-sib family, and 1243 individuals (86.2% of the total, but 98.1% of the fish with DNA amplification) were correctly assigned to their parents when tolerance for a maximum of 2 allelic mismatches between parents and offspring genotypes was allowed. Only 24 individuals (1.9%) were assigned to more than 2 parents and were therefore disqualified for further analyses. All dams gave progeny, although in highly variable numbers (124 on average, SD = 102, min = 7, max = 283). Out of 50 sires, only one displayed no contribution to the progeny, probably due to a bad sperm quality, and the contribution of the 49 sires with progeny was much more balanced than that of dams (25.4 offspring per sire on average, SD = 10.9, min = 7, max = 52). The dataset was thus appropriate to be analyzed with a sire model as proposed. The observed parental assignment rate is in the usual range for seabass, if we exclude the individuals not genotyped because of poor DNA quality [72].

### Survival and basic growth survey

Mortality was moderate during the experiment (106 individuals, 6.9% in 218 days). At tagging at 179 dph, the mean body weight of fish was BW_179_dph_ = 13.2 g (SD = 4.3) and reached BW_397_dph_ = 104.6 g (SD = 39.0) at the end of the experiment.

### Coping style

Number, proportions and mean body weight (at day of coping style) as well as the sex ratio of fish characterized as proactive and reactive during hypoxia avoidance test and the three sessions of risk taking test are reported in Table 1. Taking all tests and sessions together, we observed that 82.5% of the fish could be classified as reactive and 17.5% as proactive.

**Table 1:** Numbers, proportions, mean body weight and sex ratios of fish characterized by the hypoxia avoidance test, the three sessions of the risk taking test and the mean of the three sessions for all fish correctly assigned and with correct sex data (N = 1155 for hypoxia avoidance test, N = 1154 for RT1 RT2, RT3 and RT_mean).

	Hypoxiaavoidance test		Risktaking test							
	Hypo_status		RT1_status		RT2_status		RT3_status		RT_status_mean	
*Sex*	M	F	M	F	M	F	M	F	M	F
**Reactive (number)**	572	386	584	380	578	373	556	365	573	373
**BW (g)**	31.9	45.6	47.1	66.1	47.0	65.8	47.2	65.6	47.1	65.8
**Proactive (number)**	134	63	122	68	128	75	150	83	133	75
**BW (g)**	29.5	46.9	41.2	63.6	42.1	65.1	41.9	66.0	41.7	64.9
**Reactive (in %)**	81.02	85.97	82.72	84.82	81.87	83.26	78.75	81.47	81.16	83.26
**Proactive (in %)**	18.98	14.03	17.28	15.18	18.13	16.74	21.25	18.53	18.84	16.74

Using the hypoxia avoidance test, 83.5% of fish were characterized as reactive (at 256 dph, 572 males, BW = 31.9 g; 386 females, 45.6 g, Table 1) and 16.5% as proactive (134 males, BW = 29.5 g; 63 females, BW = 46.9 g, Table 1). Females had higher mean body weight than males (F_(1, 1151)_ = 240.1, p<0.001) but no effect of coping style and no interaction between sex and coping style were observed on body weight (F_(1, 1151)_ = 0.3, p = 0.64 and F_(1, 1151)_ = 3.4, p = 0.06 respectively).

Combining the three risk-taking test sessions, 82.2% of fish were characterized as reactive (after the third risk taking test, at 305 dph, 573 males, BW = 47.1 g; 373 females, 65.8 g, Table 1) and 17.8% as proactive (133 males, BW = 41.7 g; 75 females, BW = 64.9 g, Table 1). Mean body weights were different according to sex (F_(1, 1150)_ = 235.7, p<0.001) and to coping style as characterized by the risk taking test: reactive individuals had a higher mean body weight than proactive ones (55.0 ± 0.7 g and 50.0 ± 1.3 g respectively, F_(1, 1150)_ = 7.3, p<0.01). No interaction between sex and coping style was observed (F_(1, 1150)_ = 1.09, p = 0.30).

Proportions of individuals in each category (*i*.*e*. reactive *vs*. proactive) remained stable between tests (D_f_ = 3, χ^2^ = 6.38, p = 0.09) and were different according to the sex for the hypoxia avoidance test (Hypo_Status: D_f_ = 1, χ^2^ = 4.75, p = 0.03) but not for risk taking tests (RT1_Status: D_f_ = 1, χ^2^ = 0.88, p = 0.35; RT2_Status: D_f_ = 1, χ^2^ = 0.36, p = 0.55; RT3_Status: D_f_ = 1, χ^2^ = 1.26, p = 0.26; RT_status_mean: D_f_ = 1, χ^2^ = 0.82, p = 0.37).

### Heritability and traits correlations

Only fish correctly assigned with valid performances and identified sex were used (N = 1155) for analyses of genetic parameters. BW at the different measurements showed high heritability estimates (h^2^ = 0.47–0.59), accompanied with extremely high genetic correlations (0.87 ± 0.05 < r_A_(BW) < 1.00±0.00) as well as phenotypic correlations (0.70±0.02 < r_P_(BW) < 0.99±0.00) between successive BW values.

Heritability estimates for variables of interest in hypoxia avoidance test are reported in Table 2. These values are relatively low (0.08–0.11), except for the variable Hypo_Status (representing the hypoxia avoidance behavior) that showed a moderate heritability estimate (0.19 ± 0.10, Table 2). Hypo_Status showed very high genetic correlation r_A_ > 0.92 ± 0.44 with the other variables measured in the same test, and especially a genetic correlation of 0.98± 0.14 was estimated between Hypo_status and NBhypo. Another noticeable genetic correlation was observed between emerg_hypo and O2mean (r_A_ = 1.00± 0.10, Table 2).

**Table 2:** Heritabilities (h^2^), genetic correlations and phenotypic correlations estimated for the variables of hypoxia avoidance test using a sire models. Genetic correlations ± SE are presented above the diagonal, heritabilities h^2^ ± SE on the diagonal and phenotypic correlations under the diagonal. NE represents non estimable value due to bad model convergence.

Variables of interest	emerg_hypo	NBhypo	Hypo_status	O2mean
emerg_hypo	**0.11 (0.05)**	0.80 (0.20)	NE	0.99 (0.04)
NBhypo	0.55 (0.02)	**0.08 (0.05)**	0.98 (0.14)	0.84 (0.24)
Hypo_status	NE	0.61 (0.01)	**0.19 (0.10)**	1.00 (0.10)
O2mean	0.46 (0.01)	0.47 (0.02)	0.71 (0.01)	**0.08 (0.05)**

Heritability estimates, phenotypic and genetic correlations between the three successive risk taking test sessions are presented in Table 3. Risk-taking, measured by the binary trait RT_Status, was shown to be highly heritable (*h^2^* = 0.36–0.55, Table 3). In addition, phenotypic correlations between successive risk-taking test sessions were relatively high (0.64–0.72, Table 3) showing that behavioral response was consistent over time. Finally, very high genetic correlations between sessions were observed (0.96–0.99). The variables emerg_RT1, -2 and -3 showed moderate but significant heritabilities (0.20–0.21; Table 3). The variables NBRT1, -2 and -3 (representing activity levels) showed a low heritability (0.16 ± 0.06, for NBRT1, 0.04 ± 0.03 for NBRT2 and 0.09 ± 0.05 and NBRT3, Table 3). A weak phenotypic correlation was observed between NBRT1 and NBRT2 (0.24 ± 0.03) whereas the genetic correlation was high (0.87 ± 0.58). The phenotypic correlation between NBRT2 and NBRT3 was 0.43± 0.02 whereas the genetic correlation was 0.41 ± 0.45.

**Table 3:** Heritabilities (h^2^), genetic correlations and phenotypic correlations estimated for variables of the three risk taking test sessions using a sire models. Genetic correlations ± SE are presented above the diagonal, heritabilities h^2^ ±SE on the diagonal and phenotypic correlations under the diagonal.

	Risk taking test (sessions 1 to 3)								
Variables of interest	RT1_status	RT2_status	RT3_status	emerg_RT1	emerg_RT2	emerg_RT3	NBRT1	NBRT2	NBRT3
RT1_status	**0.55 (0.16)**	0.96 (0.06)	0.99 (0.06)	-	-	-	-	-	-
RT2_status	0.64 (0.01)	**0.41 (0.14)**	0.99 (0.05)	-	-	-	-	-	-
RT3_status	0.66 (0.01)	0.72 (0.01)	**0.36 (0.12)**	-	-	-	-	-	-
emerg_RT1	-	-	-	**0.21 (0.07)**	0.95 (0.06)	0.98 (0.06)	-	-	-
emerg_RT2	-	-	-	0.67 (0.02)	**0.21 (0.07)**	0.97 (0.04)	-	-	-
emerg_RT3	-	-	-	0.69 (0.01)	0.79 (0.01)	**0.20 (0.07)**	-	-	-
NBRT1	-	-	-	-	-	-	**0.16 (0.06)**	0.87 (0.58)	0.77 (0.29)
NBRT2	-	-	-	-	-	-	0.24 (0.03)	**0.04 (0.03)**	0.41 (0.45)
NBRT3	-	-	-	-	-	-	0.18 (0.03)	0.43 (0.02)	**0.09 (0.05)**

We then created new consolidated variables as means of each variable over the three sessions in order to assess the genetic variability of these variables (Table 4). Analyses showed important genetic correlations between the mean variables from the risk taking tests. Among them, RT_Status_mean (i.e. proactive-reactive category) showed the highest heritability (0.45 ± 0.14). The phenotypic correlation between Hypo_Status and RT_status_mean was weak (r_P_ = 0.10± 0.03), and the genetic correlation was moderate (r_G_ = 0.45 ± 0.27, Table 4).

**Table 4:** Heritabilities (h^2^), genetic correlations and phenotypic correlations estimated between the variables of hypoxia avoidance test and risk taking test using a sire models. Genetic correlations ± SE are presented above the diagonal, heritabilities h^2^ ± SE on the diagonal and phenotypic correlations under the diagonal.

Variables of interest	RT_status_mean	NBRTmean	NBhypo	Hypo_status
RT_status_mean	**0.45 (0.14)**	0.81 (0.26)	0.46 (0.27)	0.45 (0.27)
NBRTmean	0.19 (0.03)	**0.09 (0.05)**	-0.26 (0.40)	-0.26 (0.39)
NBhypo	0.10 (0.03)	-0.00 (0.03)	**0.08 (0.05)**	0.98 (0.14)
Hypo_status	0.10 (0.03)	0.01 (0.03)	0.61 (0.01)	**0.19 (0.10)**

Genetic correlations between weight and variables from the hypoxia avoidance test (Hypo_Status) were negative and stable over time (-0.62 ± 0.24 with BW at 179 dph to -0.53 ± 0.28 with BW at 397 dph). Genetic correlations between body weight and RT_Status_mean were also negative and stable (-0.28 ± 0.20 with BW at 179 dph to -0.32 ± 0.19 with BW at 325 dph).

## Discussion

In the present study, we assessed the genetic variability of body weight and coping style in European seabass, as well as phenotypic and genetic correlations between these traits. Two tests were used for measuring five traits in different contexts. An hypoxia avoidance test allowed to characterize the coping style (proactive/reactive) taking individual activity, boldness and hypoxia tolerance into account [3, 7, 16, 23] while the risk taking tests were used to address individual boldness and activity [16, 24]. We showed significant heritability of coping style measured both in the hypoxia avoidance test and during three sessions of risk taking test, and demonstrated links between growth and coping style. To our knowledge, our study is the first to assess coping style in a teleost on a very high number of individuals (N > 1000) reared in common garden, and combining parental links between individuals to assess the genetic components of behavioral traits.

### Hypoxia avoidance

The high genetic correlations between hypoxia status (tolerance vs. avoidance) and other variables derived from the hypoxia avoidance test (Number of passages between the hypoxic and the normoxic zones and the oxygen level when the fish first escapes from the hypoxic zone) make this variable (Hypo_status) the most synthetic to describe the genetic basis of an individual’s coping style [3, 7, 16, 23]. In addition, the high genetic correlation observed between activity and the oxygen threshold triggering the hypoxia avoidance suggests a common underlying genetic basis for these two traits that should probably be linked to metabolism. Indeed, this link between individual needs in oxygen and activity level is consistent with other studies on seabass [73] and on gilthead sea bream, *Sparus aurata* [74]. However, relatively weak heritability estimates were observed for these variables, suggesting a limited genetic basis.

### Risk taking

High phenotypic correlations were observed between risk-taking status and emergence time in the three sessions of the risk taking test, showing consistency over time of the behavioral responses, further reinforced by the very strong genetic correlations estimated between the risk taking test sessions. The families were therefore ranked similarly in the three sessions, i.e. risk-taking behavior is governed by the same genetic basis in the different test sessions. In addition, the observed heritability of emergence times suggests that the latency to pass from the safe to the risky chamber (i.e. decision making) was at least partially genetically determined. These results confirm the validity of the risk taking test to characterize coping style in this species.

### Link between hypoxia and risk taking

The low phenotypic correlation and the weak genetic correlations between hypoxia avoidance and risk taking tests depict that they do not assess exactly the same behavioral traits, or that they are composed at least partially of different component traits. This result confirms those of Ferrari et al., [16] showing no cross context consistency between behavioral responses of seabass using the same tests. Indeed, the hypoxia avoidance test encompasses the willingness to take risk and individual hypoxia sensibility hereby linking with an additional component through the respiratory metabolism. These results evidence complex links between boldness and hypoxia tolerance as demonstrated by Killen et al., [73] and McKenzie et al., [75], confirming that some behaviors are context specific [9]. Further, this absence of cross context consistency may be explained by the fact that correlations between behavioral responses in different contexts are generated by selection pressures partially alleviated in rearing environment and in domesticated species when compared to the wild environment [76].

Proportions of proactive and reactive fish weakly differed between sexes when characterized by the hypoxia avoidance test, but did not when characterized by the risk taking test. Contrary to what was observed in zebrafish [30] or a Poeciliid, *Brachyraphis episcopi* [77], seabass males are not bolder than females.

Coping style heritability was studied in a few teleost fish models, such as zebrafish [30], brown trout, *Salmo trutta* [31], or the cichlids *Neolamprologus pulcher* [29] and *Amatitlania siquia* [78] but our study is the first one dedicated to an economically major marine species. In European seabass, coping style traits were found to be heritable. The heritability observed in the hypoxia test (avoidance vs tolerance) was moderate while the heritability measured in the risk-taking test (risk taker vs risk avoider) was twice higher. Since variability in risk taking behavior has already been observed in many taxa, this confirms its higher importance in terms of fitness [1, 77, 79, 80]. The heritability estimated for risk taking was lower than that estimated for zebrafish (*h^2^ =* 0.76; [30]) but slightly higher than that estimated in the Honduran red point cichlid, *Amatitlania siquia* (*h^2^* = 0.37; [78]) suggesting this trait has been selected differently according to species and environmental pressures. Heritability of hypoxia tolerance in fish was never estimated before, so that we cannot compare our results with other species.

### Growth and coping styles

Genetic correlations between weight and risk taking traits showed negative values whatever the test used. This showed that proactive fish were smaller and had a lower growth than reactive ones in this population. We observed a large proportion of reactive individuals which is in accordance with a previous study [16]. This may be due to the genetic origin of our population, which is in the first stages of its domestication history. The population studied comes from individuals born in captivity but without any selection pressure and with high genetic variability, close to that observed in a wild population. A study comparing seabass from one generation of domestication *versus* a wild population showed equal growth performance [81]. However, when the coping style dimension was added, differences between phenotypes and risk taking appeared, as demonstrated by Millot et al. [82]. Indeed, Millot et al. [24] showed that in an unselected seabass population, reactive individuals had a higher mean body weight than proactive ones, whereas the opposite was observed in a population selected for fast growth. These results are confirmed by our study: reactive seabass from our unselected population had a higher mean body weight than proactive individuals. In addition, another study already demonstrated that wild reactive trout had a higher growth rate in a natural habitat [83] and by contrast numerous studies have shown that selection for growth co-selects bold and proactive individuals [51, 84, 85]. Our results are thus in accordance with the literature.

This could have numerous applications in aquaculture industry including welfare issues [12], health and disease susceptibility [10, 86] and feed efficiency [87] (reviewed in Castanheira et al. [7] and Huntingford and Adams [85]). For example, Fevolden et al. [86] suggested that selecting salmon for their coping style rather than for their specific response to pathogen (difficult to select) could enhance immune defense mechanisms on a larger spectrum, enabling a global enhancement of disease resistance. These findings all together open new perspectives for further breeding programs in teleosts. For example, the coping style approach could be used to improve fish adaptation to rearing conditions. Further studies are however needed to determine if and how selection for growth and other traits of interest may interact with behavioral traits.

## Conclusions

For the first time, we evidenced that coping style measured using hypoxia avoidance and risk taking tests was heritable in European seabass and correlated with a production trait such as growth. Importantly, heritability measured in the risk taking test shows that significant genetic gains for this trait could be achieved by selective breeding, opening a new research era for selective breeding programs aiming at enhancing the domestication process while taking into account animal behavioral responses. In addition, the high heritability of risk taking suggests its importance in terms of fitness. Overall, this study also allows a better understanding of the origin of interindividual variation in behavior.
